# Counteracting negative venous line pressures to avoid arterial air bubbles: an experimental study comparing two different types of miniaturized extracorporeal perfusion systems

**DOI:** 10.1186/s12871-015-0058-0

**Published:** 2015-05-29

**Authors:** Anas Aboud, Hendrikje Mederos-Dahms, Kai Liebing, Armin Zittermann, Harald Schubert, Edward Murray, Andre Renner, Jan Gummert, Jochen Börgermann

**Affiliations:** 1 Department of Thoracic and Cardiovascular Surgery, Heart and Diabetes Center NRW, Ruhr University Bochum, Georgstrasse 11, 32545 Bad Oeynhausen, Germany; 2 Department of Medical Technology, Friedrich Schiller University, Jena, Germany; 3 Institute of Laboratory Animal Science and Welfare, Friedrich Schiller University, Jena, Germany

**Keywords:** Miniaturized extracorporeal perfusion system, One-way-valve, Gaseous microembolism, Excessive negative venous line pressure, Systemic inflammatory response

## Abstract

**Background:**

Because of its low rate of clinical complications, miniaturized extracorporeal perfusion systems (MEPS) are frequently used in heart centers worldwide. However, many recent studies refer to the higher probability of gaseous microemboli formation by MEPS, caused by subzero pressure values. This is the main reason why various de-airing devices were developed for today’s perfusion systems. In the present study, we investigated the potential benefits of a simple **o**ne-way-**v**alve connected to a volume replacement **r**eservoir (OVR) for volume and pressure compensation.

**Methods:**

In an experimental study on 26 pigs, we compared MEPS (*n* = 13) with MEPS plus OVR (*n* = 13). Except OVR, perfusion equipment was identical in both groups. Primary endpoints were pressure values in the venous line and the right atrium as well as the number and volume of air bubbles. Secondary endpoints were biochemical parameters of systemic inflammatory response, ischemia, hemodilution and hemolysis.

**Results:**

One animal was lost in the MEPS + OVR group. In the MEPS + OVR group no pressure values below −150 mmHg in the venous line and no values under -100 mmHg in right atrium were noticed. On the contrary, nearly 20 % of venous pressure values in the MEPS group were below −150 and approximately 10 % of right atrial pressure values were below -100 mmHg. Compared with the MEPS group, the bubble counter device showed lower numbers of arterial air bubbles in the MEPS + OVR group (mean ± SD: 13444 ± 5709 vs. 1 ± 2, respectively; *p* < 0.001). In addition, bubble volume was significantly lower in the MEPS + OVR group than in the MEPS group (mean ± SD: 1522 ± 654 μl vs. 4 ± 6 μl, respectively; *p* < 0.001). The proinflammatory cytokine interleukin-6 and biochemical indices of cardiac ischemia (creatine kinase, and troponin I) were comparable between both groups.

**Conclusions:**

The use of a miniaturized perfusion system with a volume replacement reservoir is able to counteract excessive negative venous line pressures and to reduce the number and volume of arterial air bubbles. This approach may lead to a lower rate of neurological complications.

## Background

The clinical advantages of miniaturized extracorporeal perfusion systems (MEPS) are well accepted. In addition to decreasing the inflammatory response, miniaturized closed-circuit perfusion systems limit the amount of hemodilution and transfusion requirements [1]. A reduced foreign surface contact area, elimination of blood/air interface by omitting the venous reservoir, and a lower priming volume are some of the reasons for these benefits [1]. A recent study of our research group has confirmed these advantages [2]. However, we also showed that omitting the venous reservoir is associated with excessive negative venous line pressures and a significant increase in the number and volume of arterial air bubbles compared with conventional cardiopulmonary bypass (CCPB), unless specific de-airing safety procedures are introduced. Similar results were reported by Norman et al. [3]. Air bubbles may result in strokes and neuropsychological deficits [4, 5]. Excessive subzero pressures occur mainly due to suction phenomena under volume depletion [2]. When the venous line reservoir is removed in minimized systems, the perfusionist cannot counteract an insufficient venous return by adding reservoir blood, a technique commonly used with CCPB. To avoid this problem, we have developed a new **o**ne-way-**v**alve at the venous line connected to a volume replacement **r**eservoir (OVR) [6]. In the present study, we included this device in a closed perfusion system without any application of an extra air removal device. The present study aimed to investigate the effects of MEPS with or without OVR on pressure values in the venous line and right atrium. We compared the pressure differences as well as the volume and number of air bubbles in the two perfusion systems. The overall goal of this work was to increase the safety of minimized perfusion systems by reducing the risk of gaseous embolism without adding significant costs.

## Methods

### Animals

The study was carried out in 26 healthy pigs weighing 55–65 kg. The animals were kept in accordance with the German national standards on laboratory animal welfare. All experiments performed in this study were approved by the local ethics committee on animal research of the State of Thuringia and conducted at the animal laboratory of the Friedrich Schiller University Jena, Germany. The study was supported by a grant from the German Heart Foundation.

### Anesthesia protocol

The animals were pre-medicated with intramuscular ketamine (10 mg/kg body weight [BW]) and midazolam (0.5 mg/kg BW) and subsequently endotracheally intubated. Inhalation anesthesia was maintained with 1–2 % isoflurane. After an initial intravenous dose of fentanyl (0.1 mg) and pancuronium (1 mg), additional doses were administered as needed. Volume-controlled ventilation was maintained at a frequency of 14/min, a tidal volume of 6–8 mL/kg BW, and a positive end-expiratory pressure of 5 mbar. The inspired oxygen fraction was kept constant at 0.5. A right carotid artery catheter was inserted for blood pressure and blood gas monitoring. In addition, a central venous catheter was inserted through the right external jugular vein. At the end of the experiments, all animals were euthanized with a potassium chloride overdose while in deep anesthesia. This conforms with the German regulations on animal studies.

### Surgical technique

All surgical procedures were carried out under sterile conditions. After instituting hemodynamic monitoring, a sternotomy was performed. A 300 IU/kg BW heparin bolus was administered. The cardiopulmonary bypass was prepared by inserting a 40–32 F dual-stage venous cannula (Sorin, Munich, Germany) through the right atrial appendage into the inferior vena cava and a 21 F aortic cannula (Maquet Cardiopulmonary AG, Hirrlingen, Germany) into the ascending aorta. Normothermic extracorporeal perfusion was commenced and maintained for 3 hours in all experiments.

In order to investigate the differences between the two perfusion groups not only during the circulating of the pump, but also throughout real circumstances and during operative manipulations, diastolic cardiac arrest was achieved 2 hours after the initiation of the extracorporeal perfusion by clamping the ascending aorta and by instilling intermittent doses of antegrade warm blood cardioplegia (Calafiore) into the aortic root. In all pigs, the left internal mammary artery was anastomosed to the left anterior descending coronary artery. The X-clamp time was 30 min. After removal of the aortic clamp and after 10 min of reperfusion, the animals were weaned from cardiopulmonary bypass. Subsequently, the cannulas were removed and the heparin effect was reversed with a matched protamine dose. This was followed by 15 min of post-perfusion observation.

### Study design

The animals were allocated to either MEPS with OVR (designated MEPS + OVR) or MEPS without OVR (designated MEPS). On each experimental day, one or two animals were randomly operated on with the same technique. Study duration was from May 2007 until June 2009. We lost one animal in the MEPS + OVR group during initiation of anesthesia. At the end of the study, data assessment was possible in 13 animals in the MEPS group and in 12 animals in the MEPS + OVR group. Primary study endpoints were pressure values and the number and volume of air bubbles. Secondary endpoints were biochemical parameters.

### Perfusion systems

**Fig. 1:**
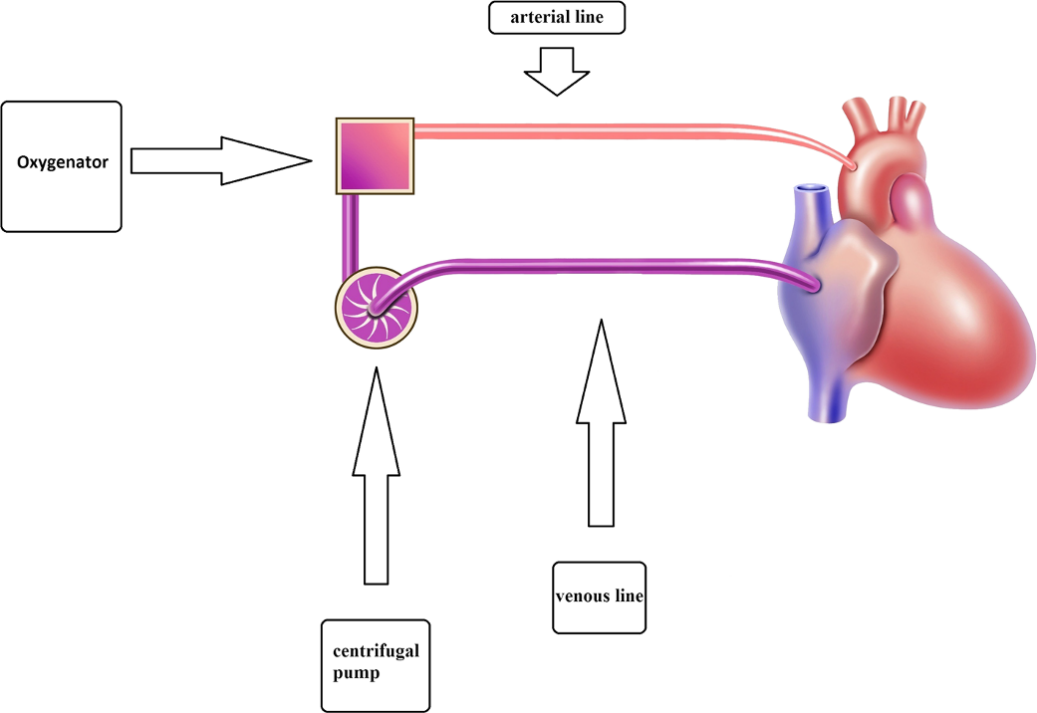
Graphical illustration of the miniaturized extracorporeal perfusion system

The MEPS circuit (Fig. 1) is a fully heparinized closed perfusion System with a high-performance hollow fiber membrane oxygenator (HILITE® 7000, MEDOS Medizintechnik AG, Stolberg, Germany) with a maximum flow rate of 7 l/min. The surface area for gas exchange is 1.9 m^2^ and the priming volume 275 ml. In addition, a centrifugal pump (DELTASTREAM DP2; MEDOS Medizintechnik AG, Stolberg, Germany) was used. A console (DELTASTREAM Driving Console, MEDOS Medizintechnik AG, Stolberg, Germany) provided manual control, adjustment and surveillance of pump function. The automatic pump speed regulator of the device was not used. Pump priming volume is 17 mL, pump speed 100–10,000 rpm, and flow capacity 0–8 l/min. We used the same heparin coated arterial filter (SENTRY, Sorin Group, Munich, Germany) with a minimum priming volume and a simple debubbling system in all experiments. Phosphorylcholine coated tubing (PVC Tubing, Sorin Group, Munich, Germany) was used. The tubing length was less than 200 cm. This setup with a total priming volume of approximately 450 ml (275 ml oxygenator, 17 ml arterial line filter, approximately 142 ml tubing) has a small blood/foreign surface contact area and results in low hemodilution. The MEPS priming fluid was made up of ringer solution (180 mL), 6 % hydroxyethyl starch (180 mL), mannitol (90 mL), and heparin (5000 IU).

**Fig. 2:**
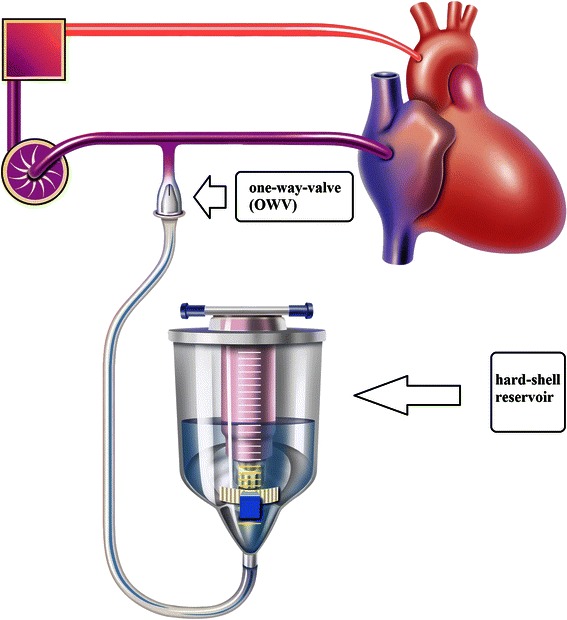
Graphical illustration of the miniaturized extracorporeal perfusion system plus volume replacement reservoir

The construction of a miniaturized extracorporeal perfusion system with automatic compensation of pressure and volume changes was patented in Germany in 2005 [7]. The perfusion system we used in the present study was based on this patent. For our study, we used different components provided by different companies. In addition to the MEPS, the perfusion system in the MEPS + OVR group included a standard hard-shell reservoir (HILITE, MEDOS Medizintechnik AG, Stolberg, Germany), which was used to substitute volume when required (Fig. 2). This reservoir was filled with Ringer solution (500 mL) and 6 % hydroxyethyl starch (500 mL). An additional component was integrated into the circuit: A one-way-valve positioned between the outflow of the reservoir and the centrifugal pump, allowing flow from the reservoir towards the pump. As a one-way-valve we use in this study the saftey silicone valve (Retroguard ® 4007100,Quest Medical, Inc.) with a priming volume of approximately 10 ml. This valve is typically used for other applications. In our system, however, the valve acts as a safeguard against excessive negative pressure by automatically opening when the pressure in the venous segment of the circuit drops below values between −75 and −90 mmHg. Thereby, volume from the primed reservoir compensates for low negative pressures [6].

In both groups, the composition of the priming volume and all equipment (oxygenator, centrifugal pump, arterial filter and tubing) was identical. There was a standard perfusion protocol for both groups with target arterial pressures of 50–60 mmHg and pump flows of 65–75 ml/kg/min BW. The volume management during the extracorporeal perfusion was controlled by the perfusionist in depending on requirement in order to have pump flow values constant in the target area. The substituation was done automatically from the connected reservoir in the MEPS + OVR group and over the central venous catheter in the MEPS group.

### Study procedures

During extracorporeal perfusion, right atrial pressure was monitored with a catheter (LAP 1751, Maquet Cardiopulmonary AG, Hirrlingen, Germany). Additionally, the venous line pressure was measured. All pressure values were digitized in 250 ms intervals using a modified analog-to-digital converter and special software developed by the Department of Medical Technology at the University of Jena.

Microbubbles were measured and analyzed with the BCC200 system (GAMPT GmbH, Zapfendorf, Germany), which is certified for clinical use. This system counts micro-bubbles and determines their size and volume, depicting the results in a histogram. The venous and arterial lines of the extracorporeal circuit were monitored with two independent sensors. In order to detect bubbles in the venous blood from the right atrium, the venous sensor was directly placed on the venous line at a point before the line connects with the perfusion system. For the arterial sensor, we chose a position on the arterial line after the arterial filter to detect air bubbles entering the aorta.

We also collected blood samples before extracorporeal circuit (t0), 10 min (t1), 60 min (t2), and 120 min (t3) after commencing extracorporeal circuit. An additional sample was collected immediately after extracorporeal circuit termination (t4). The following parameters were measured: blood gases, hemoglobin, hematocrit, lactate dehydogenase, free hemoglobin, bilirubin, interleukin 6 (IL-6), troponin I and creatine kinase.

### Statistics

Approximately 28,000 (27332 +/− 3529) arterial and venous pressure values were analyzed per animal. It was of particular interest to assess the percentage of very low negative pressures values in the right atrium and the venous line rather than mean pressure values. Therefore, values were categorized into 7 (right atrium values) and 5 (venous line values) pressure bins. The percentage of observations in each pressure group was assessed. For the statistical analysis, these values were treated as continuous variables. We also documented in this study the numbers of gaseous microemboli and their volume during extracorporeal circuit time. The pressure value percentages in each category and the air bubble numbers and volumes were analyzed using the unpaired *t*-test. A two-factor repeated-measures analysis of variance was used to assess time effects and to analyze time x treatment (type of perfusion system) interaction effects on all dependent biochemical variables. Since several biochemical parameters such as leucocytes, IL-6, and bilirubin were not normally distributed, these biochemical data were logarithmically transformed to achieve almost normally distributed data. All continuous variables were expressed as mean and standard deviation (SD). *P* values < 0.05 were considered significant. We used the statistical software package SPSS, version 20 (IBM Corp, Armonk, NY, USA), to perform the analyses.

## Results

### Primary endpoints

**Fig. 3:**
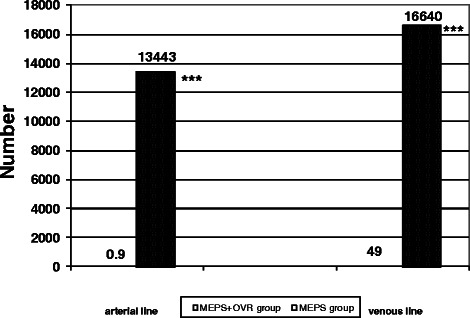
Mean number of gaseous microemboli in the arterial and venous line according to study group. ***P < 0.001 miniaturized extracorporeal perfusion system plus volume replacement reservoir group vs. miniaturized extracorporeal perfusion system group

**Fig. 4:**
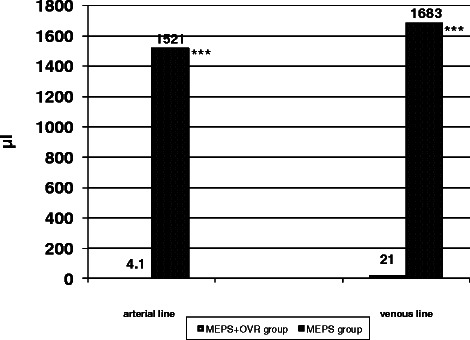
Mean volume of air bubbles in the arterial and venous line according to study group. ***P < 0.001 miniaturized extracorporeal perfusion system plus volume replacement reservoir group vs. miniaturized extracorporeal perfusion system group

The percentage of venous line and right atrial pressures in each pressure category is presented in Table 1. Compared with the MEPS group, the pressure values in the venous line as well as in the right atrium were significantly higher in the MEPS + OVR group: Approximately 80 % of venous pressure values were above −50 mmHg and only a few values were between −75 and −149 mmHg in the MEPS + OVR group. No values below −150 mm Hg were observed. Low pressure values occurred much more frequently in the MEPS group. Nearly 20 % of venous pressure values were below −150 mmHg and approximately 30 % of right atrial pressure values were below −30 mmHg in the MEPS group, 9.5 % were below −100 mmHg. The low pressure values in the MEPS group were accompanied by a much higher number of arterial and venous air bubbles than in the MEPS + OVR group (Fig. 3). In detail, the number of air bubbles in the arterial line was 13444 ± 5709 vs. 1 ± 2 (*p* < 0.001), and 16640 ± 16070 vs. 49 ± 60 (*p* < 0.001) in the venous line. In addition, the volume of the arterial and venous bubbles was much higher during MEPS than during MEPS + OVR (Fig. 4). Arterial air bubbles during MEPS and MEPS + OVR had a volume of 1522 ± 654 μl and 4 ± 6 μl, respectively (*p* < 0.001). Venous air bubbles had a volume of 1683 ± 1322 μl during MEPS vs. 21 ± 52 μl during MEPS + OVR (*p* < 0.001).

**Table 1:** Venous and arterial line pressure distribution according to study group

	MEPS + OVR group	MEPS group	*P* value
Venous line pressure (%)			
> −50 mmHg	85.7 ± 13.3	23.2 ± 20.5	<0.001
−50 to −74 mmHg	12.4 ± 11.7	37.8 ± 24.1	<0.001
−75 to −149 mmHg	1.9 ± 5.1	18.9 ± 12.0	<0.001
−150 to −299 mmHg	0	16.9 ± 11.3	<0.001
≤ − 300 mmHg	0	2.1 ± 2.1	0.006
Right arterial line pressure (%)			
>10 mmHg	3.2 ± 4.8	1.2 ± 0.9	0.664
10 to 1 mmHg	56.4 ± 7.6	17.2 ± 14.0	<0.001
0 to −9 mmHg	37.8 ± 12.0	31.7 ± 12.7	0.902
−10 to −29 mmHg	2.3 ± 0.7	18.1 ± 17.0	0.014
−30 to −99 mmHg	0.4 ± 0.3	21.1 ± 6.5	<0.001
−100 to −199 mmHg	0	9.5 ± 3.7	<0.001
≤ − 200 mmHg	0	1.2 ± 0.4	<0.001

*MEPS* miniaturized extracorporeal perfusion system; *MEPS* + *OVR* miniaturized extracorporeal perfusion system plus volume replacement reservoir group

### Secondary endpoints

**Fig. 5:**
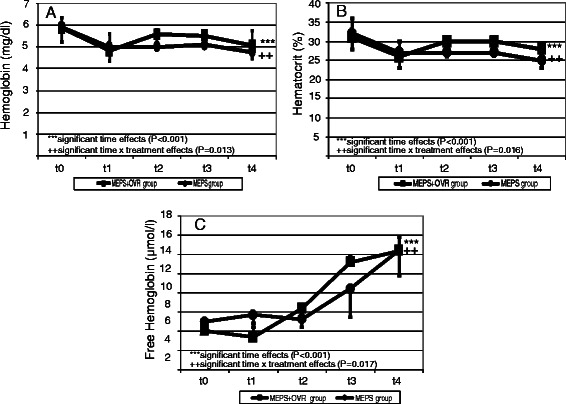
Time course of hemoglobin (**a**), hematocrit (**b**), and free hemoglobin (**c**)

**Fig. 6:**
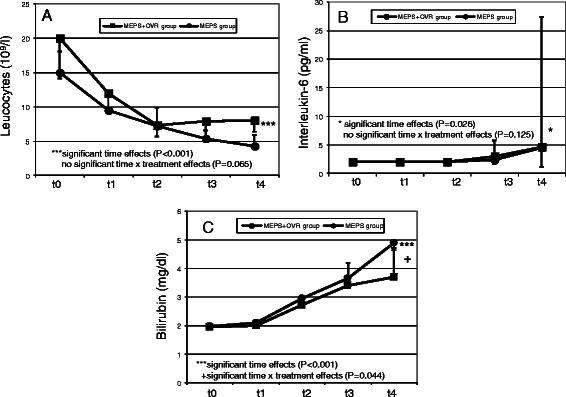
Time course of leucocytes (**a**), interleukin 6 (**b**), and bilirubin (**c**)

**Fig. 7:**
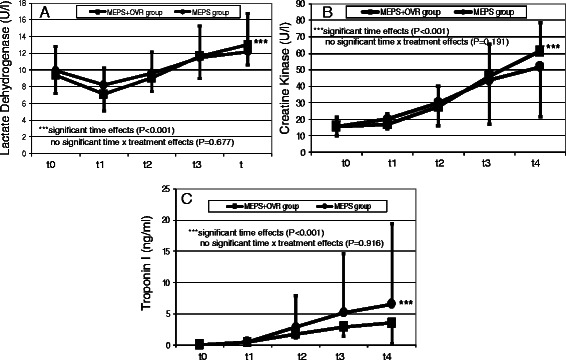
Time course of lactate dehydrogenase (**a**), creatine kinase (**b**), and troponin I (**c**)

The time courses of the measured biochemical parameters are illustrated in Figs. 5, 6 and 7. During the procedure, hemoglobin and hematocrit values decreased significantly in both study groups (*p* < 0.001). In contrast, free hemoglobin values increased markedly (*p* < 0.001). Compared with the MEPS group, hemoglobin and hematocrit values remained higher in the MEPS + OVR group (*p* = 0.0013 and p = 0.016, respectively). The increase in free hemoglobin was more pronounced in the MEPS + OVR group than in the MEPS group (*p* = 0.017). There was a time-dependent decrease in leucocyte counts (*p* < 0.001), whereas IL-6 concentrations increased during the study (*p* = 0.026). However, these changes did not differ between study groups. The increase in bilirubin was less pronounced in the MEPS + OVR group compared with the MEPS group (p = 0.044). Changes in biochemical indicators of cardiac ischemia such as lactate dehydrogenase, creatine kinase, and troponin I were comparable between study groups (*p* > 0.05).

## Discussion

This study tested a newly developed device located at the venous side of the MEPS for automatic compensation of pressure and volume changes. Upgrading the MEPS requires little effort and minimum costs. The upgrade avoids shortcomings such as the excessive negative venous line pressures and increased numbers and volume of arterial air bubbles. Importantly, the benefits of MEPS such as a reduced systemic inflammatory response, and less hemodilution and hemolysis [1] are preserved.

Many studies during the last years focused on the problem of gaseous microemboli during miniaturized extracorporeal circuit [8–11]. New de-airing devices were developed to eliminate bubbles [9]. However, studies concentrated on the elimination of already existing gaseous bubbles and not on their avoidance. We [2] and others [12] have already demonstrated that excessive negative pressures in the venous line play a major role in the production of gaseous microemboli. The observed pressure differences were primarily due to volume depletion and underline the need for an appropriate and sensitive sensor for detecting it. Standard venous pressure monitoring is not always sensitive enough to detect pressure differences [13]. Current automatic pump flow controllers act as pressure regulators, but with a delay. Under certain circumstances they are not fast enough to sufficiently counteract negative pressure nadirs. Additionally, down-regulation will sometimes lead to flow reduction and thereby to decreased perfusion. Even an experienced perfusionist and anesthesiologist handling the volume management very carefully cannot provide 100 % safety.

To address the problem of excessive negative pressures, we added a newly developed one-way-valve for automatic pressure and volume compensation to the MEPS. This valve is part of the Jena Universal Perfusion System [6]. Our study shows that the use of a one-way-valve limits excessive negative pressures, leading to a significantly reduced number and volume of gaseous emboli. This observed positive effect depends mainly on the automatic compensating mechanism of the one-way-valve. Volume lost during surgery due to diuresis or bleeding would be automatically replaced by a matched volume from the reservoir when the one-way-valve opens. Timely volume replacement avoids excessive negative line pressures, leading to comfortable operating circumstances with less stress to the anesthesiologist and perfusionist.

Arterial line bubbles passing into the aorta may cause postoperative neurological events such as stroke or transitory psychotic syndrome [4]. Helps et al. [14] reported in an experimental study in rabbits that arterial air emboli of 25 μl caused only transient changes in the cortical somatosensory evoked response, whereas bubbles > 400 μl caused prolonged adverse effects. We therefore believe that every effort should be taken to avoid air bubbles and to increase the safety of all perfusion systems. Due to a reduced foreign surface area and avoidance of blood-air contact, MEPS is associated with lower systemic inflammatory response than CCPB [1]. Pro-inflammatory cytokines such as IL-6 increase in response to many major surgical procedures as well as to cardiopulmnary bypass [1]. In our study, circulating IL-6 levels did not differ between study groups. This may be due to the consistently low levels of this proinflammatory cytokine from t0 to t3 and the large standard deviation at t4. It is also noteworthy that biochemical indicators of cardiac ischemia were identical in both groups. Although volume substitution was done automatically during one-way-valve use, levels of hemoglobin and hematocrit stay slightly higher than without one-way-valve. This may be due to easier and more optimized volume management. To avoid suction phenomenon at the venous side during miniturized perfusion system, there might be the tendency to substitute more volume as needed, until the target pump flow is achieved. Free hemoglobin and bilirubin differences between groups were small and probably of minor clinical relevance.

The present study has limitations. Despite its clear results, it remains an experimental investigation with a small number of cases. Clinical endpoints like postoperative neurological or neurocognitive complications were not considered. Randomized controlled trials are still needed to compare the effect of different perfusion systems on neurological outcome.

## Conclusions

Miniaturized perfusion systems can become safer by adding a simple one-way-valve -connected to a volume replacement reservoir- to the venous side for automatic pressure and volume compensation. With this novel approach, advantages of mini-systems such as less systemic inflammatory response are preserved and disadvantages such as excessive subzero pressures and gaseous microemboli can be avoided.

## Abbreviations

BW: Body weight
CCPB: Conventional cardiopulmonary bypass
IL: Interleukin
MEPS: Miniaturized extracorporeal perfusion systems
OVR: One-way-valve volume replacement
